# Development of an Electrochemical Immunosensor for Fumonisins Detection in Foods

**DOI:** 10.3390/toxins2040382

**Published:** 2010-03-24

**Authors:** Mohamad Kamal Abdul Kadir, Ibtisam E. Tothill

**Affiliations:** Cranfield University, Cranfield Health, Cranfield, Bedfordshire, MK43 0AL, UK; Email: m.abdulkadir.s06@cranfield.ac.uk

**Keywords:** fumonisins, mycotoxins, screen-printed gold electrode, electrochemical immunosensor

## Abstract

An electrochemical affinity sensor for the determination of fumonisins mycotoxins (Fms) using monoclonal antibody modified screen-printed gold electrode with carbon counter and silver-silver chloride pseudo-reference electrode is reported in this work. A direct competitive enzyme-linked immunosorbent assay (ELISA) was initially developed, exhibiting a detection limit of 100 µg·L^-1 ^for fumonisins. This was then transferred to the surface of a bare gold screen-printed electrode (SPGE) and detection was performed by chronoamperometry, monitoring the reaction of 3,3’,5,5’-Tetramethylbenzidine dihydrochloride (TMB) and hydrogen peroxide (H_2_O_2_) catalysed by HRP at −100 mV potential *vs.* onboard Ag-AgCl pseudo-reference electrode. The immunosensor exhibited detection limit of 5 µg·L^−1^ fumonisins with a dynamic range from 1 µg·L^−1^–1000 µg·L^−1^. The sensor also performed well in extracted corn samples.

## 1. Introduction

In recent years, fumonisins which are mycotoxins produced by a variety of fungi of the *Fusarium* genus have become one of the most widely researched areas of mycotoxins contamination in food. These toxins were first isolated from a culture of *F. verticillioides* by Gelderblom and co-workers [1]. Fumonisins are considered as natural contaminants of cereal grains worldwide and are mostly found in corn and corn products [2,3,4,5]. The major compounds of fumonisins are B_1_ (FB_1_) and B_2_ (FB_2_), with more than eleven structurally related compounds has been discovered to date. However, fumonisin B_1_ is the most abundant and toxic of this family of mycotoxins. Fumonisins have been linked with induce equine leukoencephalomalacia in horses and other equids [6], pulmonary edema in swine and pigs [7] and esophageal cancer in humans [8,9]. Also according to Wang *et al*., [10], fumonisins have been linked with various diseases associated with liver and kidney toxicity and carcinogenicity and immunosuppression. Therefore, the US Environmental Protection Agency (EPA) classified fumonisins as category 2B carcinogens [11]. The U.S. Food and Drug Administration (FDA) have recommended a maximum level of fumonisins (2–4 mg·L^−1^) on the hazard of animal studies for the protection of human consumption. As evidence mounts worldwide implicating fumonisins in human and animal diseases, increasing efforts to develop sensitive, selective, rapid and versatile procedures for detecting fumonisin levels in foods have emerged. 

Normally, a program of monitoring and surveillance of fumonisins level is therefore necessary to ensure safe exposure, especially in the food and feed supply. Therefore, any analytical method used must be able to detect fumonisins at least down to the specified regulatory level. Fumonisins detection techniques are typically based on chromatographic separation, quantification and identification. This includes methods such as thin-layer chromatographic (TLC) [12], liquid chromatography (LC), liquid chromatography - mass spectrometry (LC-MS) and post-hydrolysis gas chromatography (GC) and GC-MS. All of the methods above require sample extraction and clean-up steps before the analysis [13,14]. However, some of these methods are very sophisticated, expensive, time consuming and are generally unsuitable for rapid, on site or wide–scale monitoring programs. Also many chromatography methods used for mycotoxins require a form of chemical derivatization of the sample before detection is possible (with the exception of using mass spectrometry).

The growing number of mycotoxin to be controlled worldwide requires that rapid and reliable methods must become available for risk assessment and management of foods and feeds and to comply with the legislation. Because of the necessity, a number of fumonisin antibodies have been developed to allow for a rapid analysis by immunoassay techniques. Therefore, immunoassay methods for fumonisins analysis using polyclonal and monoclonal antibodies have been developed in the past two decades because of their, simplicity and selectivity [11]. Hence, on-site immunochemical techniques such as dipstick [15] immunochromatography [16], immunofiltartion [17], enzyme–linked immunosorbant assays (ELISA) and immunosensor techniques [18,19] are gaining interest for mycotoxin detection. 

Nowadays, immunosensor techniques with different sensing receptors and transducers are considered as a major development in screening methods for use in fumonisins determination. The possible advantages of immunosesnor over conventional ELISA methods are; an increase in sensitivity and decrease in low detection limit; cost effective simple to use, decrease amount of expensive reagent and portable devices with digital signal outcome [20]. In case of fumonisins detection, many immunosensors have been reported using optical sensing principle such as surface plasmone resonance [18] and fiber optic [21,22,23]. The lower detection limit of FB_1_ obtained were 50 µg·L^−1^[21] and 10 µg·L^−1^[23] using SPR and fiber optic immunosensors respectively. However, there are no reported literatures on the use of screen- printed electrodes (SPE) for fumonisins detection.

In this paper, we report on the development of an electrochemical immunosensor for fumonisins detection using a screen-printed gold electrode (SPGE). The sensor is as an attractive alternative to immunoassay techniques and also to the common use of carbon electrode in biosensors developments. Electrochemical immunosesnors have been proven to be very sensitive analytical tools obtaining low detection limits and offer reduced instrumentation costs compared to their optical counterpart [24]. The developed sensor is based on a competitive reaction between free fumonisins in the sample and a fumonisin- horseradish peroxidase conjugate, for an immobilised monoclonal anti-fumonisins antibody. Chronoamperometry was used as the electrochemical detection method for the signal generated by the use of TMB/H_2_O_2_ to ascertain the concentration of HRP on the sensor and consequently the concentration of fumonisins in the sample. The immunosensor was optimised regarding the immobilization of the ELISA reagents on the surface of the gold electrode. The optimum conditions were then employed to monitor the concentration of fumonisins in buffer solutions and then in extracted samples. The electrochemical detection involves chronoamperometry and the use of a TMB/H_2_O_2_ substrate catalysed by horseradish peroxidase which has been widely applied with screen- printed immunosensors [25,26,27,28].

## 2. Results and Discussion

### 2.1. Optimization of the ELISA method

**Figure 1:**
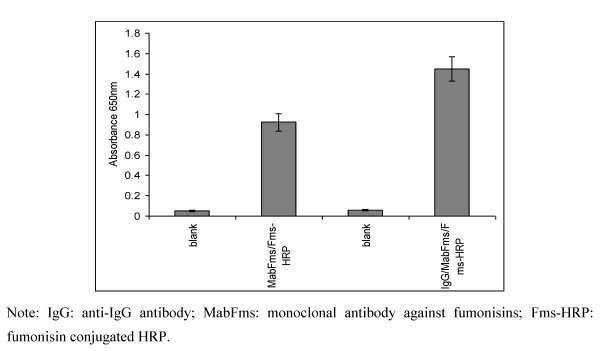
Comparison of different immobilization protocols of the monoclonal anti-fumonisin in a direct non competitive format using either non-coated (immobilized monoclonal anti-fumonisins, 1:50 µg·mL^−1^, blocked with 1% PVA) or pre-coated wells(immobilized anti-antibody IgG, 20 µg·mL^−1^, blocked with 1% PVA then monoclonal anti-fumonisins, 1:50 µg·mL^−1^) before continue with Fms-HRP from Veratox kit (1:10). Error bar = SD, n = 3.

A spectrophotometric competitive enzyme-linked immunosorbent assay (ELISA) for fumonisins detection was first developed and optimized before moving the assay to the electrochemical transducer. The tests were performed in a microwell plate based on direct method. A checkerboard titration method was used to optimize the reagents concentrations (coating antibody, monoclonal antibody and fumonisin- HRP conjugate) followed by optimization of the assay conditions (incubation times and temperatures). The use of anti-IgG to pre-coat solid faces surfaces before immobilising the anti-capture antibody has been reported to increase the detection limit of aflatoxin M_1_[29]. Therefore, to maximize the ELISA signal the use of pre-coated microtitre wells was investigated in this study. The results achieved (Figure 1), show that the use of a pre-coated wells produced a much greater signal than noncoated wells.

The increase in the signal indicates a better binding orientation of the antibodies in the assay and therefore it was applied in future tests and sensor development. The use of 1% PVA in PBS as a blocking solution in the assay produced the optimal results for non-specific binding when compared to other blocking agents (Bovine serum albumen or Gelatin) and therefore, it was used in further tests (data not shown). These results were similar to those reported by Warwick [30]. The optimal concentrations and conditions for the ELISA assay using the direct competitive format were found to be; pre-coat the wells with Anti-IgG antibody (20 µg·mL^−1^, overnight, 4 °C); block using 1% PVA in PBS (1 hour); immobilize the monoclonal anti fumonisins on the coated and blocked surface (1:50 dilution, 2 hours at 37 °C) and lastly the use of a fixed amount of Fms-HRP (1:10 dilution, 30 minutes, 37 °C).

**Figure 2:**
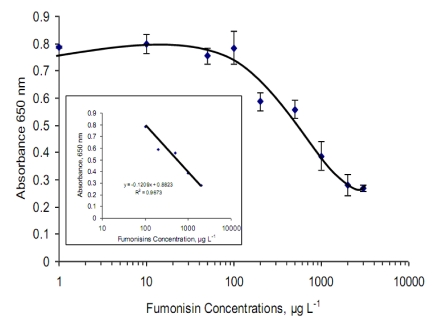
Standard curve for the detection of fumonisin using a spectrophotometric ELISA assay. Signals were obtained using a precoated wells with anti-antibody IgG (20 µg·mL^−1^), blocked with 1% PVA then anti-fumonisin antibody (MabFms) (1:50 dilution) before continue with competition Fms-HRP from Veratox kit (1:5) and free Fms (0 to 3000 µg·L^−1^). Error bar = standard deviation, n = 3.

Utilizing the derived optimal concentrations and conditions for the competitive assay, a calibration curve for fumonisins was then carried out with free (0–3000 µg·L^−1^) and HRP labelled fumonisins (Fms) in buffer, mixed and added to the wells where they compete for the anti-fumonisin active sites (Figure 2). The calibration curve was fitted using ‘non-linear regression plot’ [31]. A dynamic range from 100 to 2000 µg·L^−1^ (R^2 ^= 0.967) with an LOD of ~100 µg·L^−1^, CV = 9.3% was achieved using the linear section of the curve to calculate the data. 

### 2.2. Optimization of the immunosensor

For the immunosensor development an electrochemical transducer was chosen for the detection system, since no previous literatures have been reported for fumonisin analysis using an electrochemical immunosensor. Recent literature have shown the use of electrochemical immunosensor for a range of mycotoxin detection such as aflatoxin B_1_ [32], aflatoxin M_1_ [26,29] and ochratoxin A [33] with good detection limits below the required European Union (EU) legislation. 

Therefore, an electrochemical immunosensor system was developed in this work by transferring the ELISA assay to the gold screen-printed electrode surface, where the horseradish peroxidase (HRP) enzyme label activity is detected using chronoamperometry. TMB/H_2_O_2 _were chosen as the mediator/substrate system. (See Schematic diagram below).

**Scheme 1:**
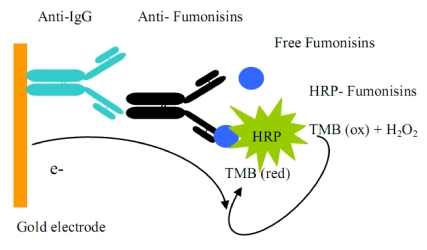
A schematic diagram of the electrochemical sensor

According to Parker & Tothill, [26] and Volpe *et al*., [34] the use of TMB as the mediator with H_2_O_2 _has greater electrochemical detection properties than other mediators system used with HRP enzyme. Therefore, in order to obtain the optimum applied potential, chronoamperometry detection for HRP using TMB/H_2_O_2_ substrate was investigated for the DuPont gold screen-printed electrode. Our previous work [25] has shown that the optimal potential using the same substrate/mediator system with an Ercon (Inc. USA) gold screen-printed electrode (R-464 (DPM-78)) was at −200 mV versus Ag/AgCl. Therefore, selecting the correct potential to use with the DuPont gold ink (BQ331 gold) on the screen-printed electrode was also investigated. The technique was evaluated by the ratio of the signal (S) current to background (B) at a constant step potential range from −600 to +600 mV. The results (data not shown) indicated that the maximum value of signal/background ratio for the best potential was at −100 mV. Therefore this potential was selected for the electrochemical detection system using the DuPont gold ink as the working electrode. 

**Figure 3:**
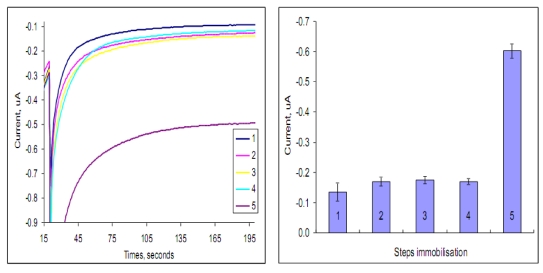
Chronoamperometric response of the different immobilisation steps of direct non competitive format using different gold electrode including 5 steps: (1) bare electrode; (2) Anti-IgG; (3) Anti-IgG/PVA/MabFms; (4) Anti-IgG/PVA/Fms-HRP and (5) Anti-IgG/PVA/MabFms/Fms-HRP using TMB/H_2_O_2_ as the enzyme substrate (n = 3).

The effect of the different immobilization steps taking place for fumonisins detection on the electrochemical signal as a background and assay signal was also investigated. Figure 3, show the signals achieved from; (1) gold bare electrode; (2) precoating of anti-antibody IgG (anti-IgG); (3) anti-IgG, blocking with PVA and MabFms (anti-IgG/PVA/MabFms); (4) anti-IgG/PVA/Fms-HRP and (5) anti-IgG/PVA/MabFms/Fms-HRP. This is an important characterization step to evaluate the performance of different reagents adsorption as well as to study the effect of the adsorbed reagents on the electron transfer characteristics of the sensor. In this study TMB/H_2_O_2_ was also used as the enzyme substrate for the measurement of the enzyme activity on the electrode surface at a potential −100 mV. Figure 3, indicate that the reagents immobilization did increase the background signal slightly but had a minimal effect on the electrochemical characteristic of the sensor and was able to detect the signal achieved from the activity of the enzyme label at high sensitivity. According to Fu, [35] conducting this type of test is important to ensure that the enzyme label is still active and the electrode surface is not blocked due to the chemical modification of the sensor surface. 

**Figure 4:**
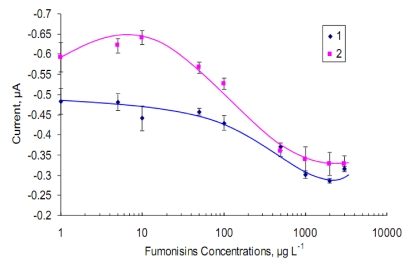
Standard curve of electrochemical competitive immunoassay on the screen-printed gold electrode for fumonisin detection: (1) the curve by direct mixing of free Fms and Fms-HRP on the gold working electrode surface; (2) the curve via a pre-incubation by adding the free Fms for 30 min before the Fms–HRP conjugate. Gold working electrodes were pre-coated with Anti-IgG antibody (20 µg·mL^−1^) and coated with MabFms (1:50 dilution) before competitive binding. The chronoamperometry, applied potential: −100 mV; chromogen/substrate of HRP: substrate; 5 mM TMB + 0.075% H_2_O_2 _in citrate-phosphate buffer pH 5.2. Error bar = standard deviation, n = 3.

A standard curve was then conducted on the screen-printed gold electrode similar to that used for the ELISA assay. The competition system was employed within free fumonisins concentrations at different range (0 to 3000 µg·L^−1^) and Fms-HRP (1:10) through optimal condition and concentrations of reagents as described previously. The standard curves of competition binding of antigen by two different assay protocols were obtained as shown in Figure 4. The first protocol gave a working range of 50 to 2000 µg·L^−1^ and LOD at ~50 µg·L^−1^, when free and labelled fumonisins were mixed together and incubated on the working electrode surface. The second protocol, a pre-incubation step was introduced (30 minutes) for the free fumonisin in the sample on the electrode surface before the addition of the HRP fumonisins conjugate. A working range of 10 to 1000 µg·L^−1^ and an LOD of ~10 µg·L^−1^ were achieved using this second assay system. The current response is inversely proportion to the fumonisin concentrations. The second method of analysis showed an increase in the assay sensitivity with a lower LOD (CV = 9.6%). Therefore, this method was used in further assays. However, two point’s needs to be considered here, the first is that this method adds an extra step to the assay protocol and the second is that by changing the incubation time and optimize it further the method without the pre-incubation step (1, Figure 4) may prove to be adequate for fumonisin analysis at the required legislative limit. The data shown in Figure 4 were carried out with two different sets of sensors. The pre-incubation tests were conducted using newly fabricated sensors, and therefore they show a higher current density. These data suggest that the reliability of the electrochemical immunosensor with the direct competitive ELISA format were suitable for fumonisins analysis at the level much lower than that required by the EU legislation (2000 to 4000 µg·L^−1^). 

### 2.3. Cross-reactivity study

**Figure 5:**
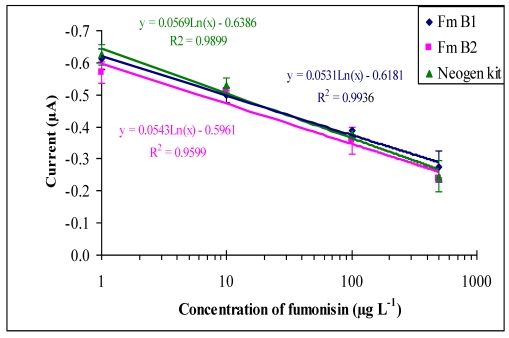
Cross reactivity of MAbFms against the standard solution of fumonisin provided in the Neogen Elisa kit, FmB_1_ and FmB_2_. Direct competitive of Fms immunosensor response on a modified gold working electrode by chronoamperometry at potential −100 mV and using a mixture of TMB (5 mM) and H_2_O_2_ (0.075%) as a substrate. Screen printed gold electrode were coated with anti-IgG (10 µg·mL^−1^), blocked with 1% PVA followed by MAbFms (1:50) then continue with Fms (1–1000) for 30 minutes preincubation times before adding of Fms-HRP (1:10). Error bar = standard deviation, n = 3.

The specificity of the developed immunosensor for the detection of Fumonisin B_1 _(FB_1_) and Fumonisin B_2_ (FB_2_) was investigated in this system. The purpose of the test is to determine the specificity of the sensor for these two structurally related toxins (Figure 5). 

Figure 5, indicate that the monoclonal anti-fumonisins used in the development of the immunosensor had similar specificity for both toxins and showed similar cross-reactivity for the two structurally relater toxins. The reactivity of MabFms with different fumonisin analogs provides an insight as to the antibody ability to bind to fumonisins toxins. A standard fumonisin solution provided by the Neogen ELISA kit was also tested using the sensor and sensor response to this solution was also compared to response achieved using FB1 and FB2 toxins. Furthermore, the sensor was also tested for the detection of aflatoxin B_1_ and Ochratoxin A, which are non structurally related toxins, and gave negative results which are below the 1% cross reactivity (data not shown). This indicates the specificity and sensitivity of the developed sensor for fumonisins detection and analysis.

### 2.4. Optimized immunosensor calibration curve

**Figure 6:**
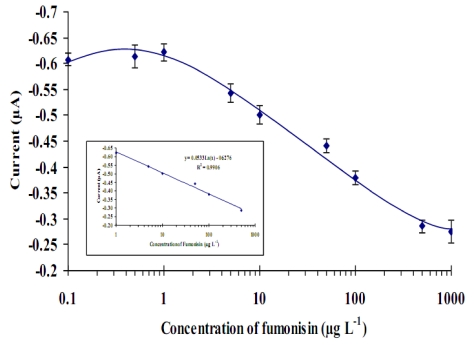
Calibration curve of direct competitive immunosensor for fumonisins analysis on thiol modified gold electrode surface. Measurements conducted by chronoamperometry at −100 mV, TMB (5 mM) and H_2_O_2_ (0.075%) as a substrate. Modified screen- printed gold electrode were coated with anti-IgG (10 µg·mL^−1^), blocked with 1% PVA followed by MAbFms (1:50) then continue with Fms (0.01–1000) for 30 minutes pre-incubation time before adding the Fms-HRP (1:5). The curve was fitted by non linear regression, with the insert showing the liner section of the curve. Error bar = SD, n = 3.

The calibration curve for the immunosensor was conducted using all the optimized parameters for the competitive reaction on the gold sensor surface with a low range of fumonisins standard solution (0.01 to 1000 µg·L^−1^ ) and a 30 minutes pre- incubation step (toxins solutions incubated on the monoclonal antibody immobilized on a thiol modified gold surface). The results are shown in Figure 6. 

Figure 6, show that the decrease in current response was proportional to the fumonisin concentration in the range of 1 to 500 µg·L^−1^. The linear regression equation is µA = 0.053 × C_[Fms]_ − 0.63 with a limit of detection (LOD) of ~5 µg·L^−1^ (estimated to be 3× the standard deviation of blank-dose signal, n = 3, R^2 ^= 0.991). In this case, the LOD obtained in this final optimized assay system with the pre-incubation step was lower than reported previously. This sensor proved to be very sensitive and for low level of fumonicins detection in buffer solutions. Using this device and technique with highly contaminated food samples (above 500 µg·L^−1^) may require the sample to be diluted to obtain the toxin concentration. 

### 2.5. Sensor response in extracted corn samples

To test the performance of the sensor in real sample matrix and conduct a recovery test for fumonisin, ground corn samples were spiked with various concentrations of mixed FB_1_ and FB_2_. The samples were first extracted using methanol/water mixture (70:30) and then either first used without any further treatment or the second were cleaned and concentrated using a clean-up procedure (C-18 SPE column). Samples were then dried and resuspended in PBS buffer and quantified using the developed electrochemical immunosensor assay. Other none spiked corn samples were also extracted and determined in order to evaluate possible matrix effect compared to fumonisins in buffer samples. For non spiked samples background signal, the current archived was found to be similar to the signal normally achieved in buffer (data not shown). This indicates that there is minimum matrix effect on the background electrochemical signal of the immunosensor by following the developed procedure. In spiked corn samples, the recovery of fumonisins was determined from the analyzed concentrations of fumonisins in the corn samples, compared to the expected values spiked to the sample. Using a calibration curve of fumonisin in a non spiked corn extract and C-18 cleaned samples, the obtained recovery results for spiked samples were calculated and these are shown in Table 1. 

**Table 1:** A comparison results of corn samples spiked with mixed Fumonisins (FB_1_ and FB_2_), and using two different extraction methods, the samples were analysed using the electrochemical immunosensor.

Spiked Corn (µg·kg^−1^)	Without clean-up			Clean-up using C-18		
	Found (µg·kg^−1^)	% R	SD	Found (µg·kg^−1^)	% R	SD
Non Spiked	<1		6.12	<1		4.08
50	25.05	60.1	9.43	50.85	101.7	7.6
250	144.65	69.86	5.54	190.1	76.04	8.49
500	410.9	82.18	5.96	560.3	112.06	4.61
2500	2055.8	82.23	3.61	2616.05	104.64	3.87

When the two different extraction procedures were compared in terms of recovery, different percentage recoveries were found. The average of recovery result for the extraction method without a clean-up step (rapid extraction technique) was much lower than the average recovery with a clean-up and concentration step (using C-18 SPE) (Table 1). The lower concentration of spiked fumonisins (50 and 250 µg·kg^−1^) obtained less recovery compared to higher concentration (500 and 2500 µg·kg^−1^). This may suggest that substances in the samples containing low fumonisins inhibited the toxin signal response. By using a clean-up procedure (C-18 SPE column) which will help in removing these substances, the recovery of fumonisins have increase due to their removal from the sample extract. According to Muscarella *et al*. [36], and in agreement with regulation 401/2006/EC, that recovery in the range of 60–120% for fumonisins is expected for samples containing ~500 µg·kg^−1^. From the achieved results conducted in this study, the use of a clean-up procedure is recommended to improve fumonisins recovery especially at the low contamination level. Also conducting a standard curve for fumonisins using unspiked corn samples extracted and cleaned using C-18 gave very comparable results to fumonisin in buffer analysis (data not shown). The performance of the immunosensor and the results achieved from analysing the corn samples indicate its useful application for the analysis of fumonisins in corn samples. This indicates the developed SPGE immunosensor using electrochemical detection is rapid and reliable for corn samples analysis. 

## 3. Experimental Section

### 3.1. Reagents and solutions

Mouse monoclonal anti-fumonisin antibody (MabFms) was purchased from Abcam Ltd. (Cambridge, UK), fumonisin- HRP conjugate and a chromogen/substrate solution was from Veratox kit from Neogen Corporation (UK). Affinity purified, anti-mouse IgG (H+L) from goat was obtained from Pierce Ltd. (UK). Fumonisins standards (Fumonisin B_1_ and Fuminisin B_2_), polyvinyl alcohol (PVA), 30% hydrogen proxide (H_2_O_2_), 3,3’,5,5’-tetramethylbenzidine dihydrochloride (TMB) (powder), Tween-20, potassium chloride (KCl) and all other buffer reagents were purchased from Sigma-Aldrich Co. Ltd. (UK). TMB solution for ELISA tests was purchased from Insight Biotechnology (UK). Organic solvents (methanol and acetonitrile) were HPLC grade, purchased from Fluka (UK). Sodium carbonate buffer (CB), 100 mM, pH 9.6, was prepared by dissolving Na_2_CO_3 _(1.59 g) and NaHCO_3 _(2.93 g) in in 1 L distilled-deionised water and the pH adjusted to 9.6. Phosphate buffered saline (PBS), 10 mM, pH 7.4 was prepared by dissolving 5 buffer tablet in 1 L distilled-deionised water. PBS-T solution was prepared by adding 0.05% tween-20 (v/v) to PBS buffer. Citrate-phosphate buffer, 50 mM, pH5.5 was prepared by dissolving one buffer tablet in 100 ml of distilled-deionised water. TMB substrate solution was prepared by dissolving 1 mg of TMB in 150 µL of distilled deionised water. The Fumonisins (Fms) standard stock solutions were prepared by dissolving 1 mg in 1 mL methanol (HPLC grade) and stored at −18 °C in tightly capped and dark bottle. When needed the stock solutions were diluted in PBS, 10 mM, pH 7.4 to the concentration range of 0–3,000 µg·L^−1^ and used for ELISA and immunosensor tests.

### 3.2. Fabrication of screen-printed gold electrodes

Screen-printed gold electrodes (SPGE) consisting of gold working electrode, carbon counter electrode and silver–silver chloride pseudo-reference electrode were fabricated using a procedure similar to that described in details by Noh and Tothill [37]. The electrodes used in this work were printed using the screen-printing facilities at DuPont Ltd. (Bristol, UK). The printing pastes used were 107255-135E carbon, BQ331 gold, 5874 Ag/AgCl and 5036 blue encapsulant, all inks were from DuPont Ltd.). The SPGE used in this work, consisting of a gold working electrode with a 5 mm diameter giving a 19.6 mm^2 ^planar area, printed on a graphite ink layer (dried at 120 °C, 30 min). All electrodes were then tested using a multimeter before use. The sensors edge connector was purchased from Maplin Electronics Ltd. (Milton Keynes, UK).

### 3.3. Immunoassay developments

ELISA tests were first developed using a micro well polystyrene plates, MaxiSorp (Nunc Immuno), purchased from Fisher Scientific (Loughborough, UK). An incubator/shaker HT from Labsystem iEMS were used for temperature control incubation of every step of the immunoassay reaction. Spectrophotometric analysis of colour developed was performed using a BMG Fluorstar galaxy ELISA plate reader (Aylesbury, UK).

#### 3.3.1. Direct immunoassay test

A checkerboard ELISA procedures were carried out to evaluate and optimise the concentrations of the assay reagents used in the test. Anti-mouse IgG (H+L) was first used to pre-coat the ELISA plate at a concentration range from 0 to 50 µg·mL^−1^. Anti-Fms (primary) monoclonal antibodies were immobilised on the pre-coated plates using a concentration range of 1:25 to 1:5000 dilutions) and Fms-HRP conjugate using a concentration range of 1 to 1:50 dilutions. The tests were performed in a 96-well microplate according with a direct ELISA format based on the method described by Anna *et al*. [38]. 

#### 3.3.2. Competitive assay

The competitive assay was carried out using the following procedure; the microtiter plate was pre-coated with anti-mouse IgG antibody (20 µg·mL^−1^, 50 µL/well) in 0.1 M carbonate buffer pH 9.6, for 18 hours (overnight) at 4 °C, followed by washing twice with 150 μl/well phosphate buffered saline containing Tween-20 (PBS-T) and once with PBS alone. The plate was then blocked with 1% PVA (50 µL/well) and incubated for 1 hour at 37 °C. After washing as above, the plate was coated with anti-Fms monoclonal antibody (1:50 dilution, 50 µL/well) in PBS (v/v) for 2 hours incubation at 37 °C, followed by washing. The competition solution was prepared by mixing (50 µL/well) of free fumonisin B_1_ (0–3000 µg·L^−1^) in PBS (v/v) with fixed dilution of a solution of fumonisin-HRP conjugate (1:5 dilution) in PBS (v/v). The competition reaction was allowed to proceed for 30 minutes at 37 °C and then rinsed with PBS-T (twice), followed by PBS (once). Finally, the absorbance was measured by the addition of TMB substrate solution (50 μL/well) to each well and incubated at room temperature for 15 minutes before added a 25 µL of stop reagent (H_2_SO_4_) and then measuring at 650 nm using the plate reader.

### 3.4. Electrochemical immunosensor

#### 3.4.1. Electrochemical measurements

Electrochemical procedures were conducted using a computer controlled four channel Autolab electrochemical analyser multipotentiostat (Eco Chemie, Utrecht, The Netherlands) throughout which allows the simultaneous detection of four sensors. Data capture was through the supplied GPES version 4.9 software installed onto a PC. The screen-printed electrodes were connected to the Autolab, using an in house fabricated connector from a PCB edged IDC socket, aluminum instrument box, ribbon cable and 4 mm cable sockets. The individual components were purchased from Maplin Electronics (Milton Keynes, UK). For the C.V. scans a 100 μL drop of 5 mM potassium hexacyanoferrate (III) in 0.1 M KCl was placed onto the electrode and each electrode was disposed of after each scan. The scanning range was from −0.3 to +0.8 V at a rate of 50 mVs^−1^ relative to the on board Ag-AgCl reference electrode. For samples analysis each measurements was carried out in triplicate using a new strip in a non-deaerated and unstirred solution. For the selection of optimal constant potential for the enzymatic reaction (TMB-H_2_O_2_-HRP) system, choroamperometry was conducted with bare screen-printed gold electrode with buffer solution (50 mM citrate-phosphate buffer, pH 5.5, in 0.1 M KCl), substrate (5 mM TMB, 0.07% H_2_O_2_)with fumonisin- HRP conjugate in citrate-phosphate buffer –0.1 M KCl. Step amperometry was conducted at a range of potential from +600 mV to −600 mV within 600s. 

#### 3.4.2. Direct competitive assay

A 10 µL of anti-antibody (anti-mouse IgG (H + L)) primary layer (capture species) in CB, pH 9.6 were placed on the gold working electrodes then kept overnight at 4 °C. A 20 µL of 1 % PVA (w/v) in PBS was then used to block the electrode surface for 1 hour at 37 °C. Then, 10 µL of anti-Fms antibody capture was coated on the electrode for 2 hours at 37 °C. The electrodes were then ready for fumonisins analysis using the competitive assay. A standard curve for fumonisin was then constructed on the gold electrode surface by mixing (v/v) fumonosin-HRP conjugate with free fumonisin in buffer (0–3000 µg·L^−1^ range) and placing a 10 µL of this mix on the electrode surface for the competitive assay (incubation for 30 minutes at 37 °C). The electrodes were washed between steps by rinsing using PBS-T (twice) followed by PBS (once). A substrate solution was then placed on the electrode surface and the signals measured using amperometric analysis as described above.

#### 3.4.3. Calibration plot and interpretation of result

ELISA and immunosensor calibration curves were fitted by non-linear regression using the following four parameter logistic function [31]:

 F(*x*) = (*a* – *d*)/[1 + (*x*/*c*)^*b*^] + *d*

Where parameter *a* and *d* are the asymptotic maximum and minimum value of the calibration curves, respectively, *x* the concentration at the EC_50_ value, *c* the analyte concentration and *b* is the hill slope. EC_50_ is an effective concentration for 50% value.

The limit of detection (LOD) was defined as the concentration of toxin equivalent to three times the value of standard deviation (σ). This was calculated based on the following equation:

LOD = *x* [*a – d*/ (*a – d*) − 3σ]^−1/*b*^

Where σ is the standard deviation of the zero value.

### 3.5. Corn samples analysis

#### 3.5.1. Extraction without clean-up

Samples preparation and extraction were conducted following the procedure as described by the Veratox ELISA kit (Neogen Corporation, UK). A corn sample was first ground and then mixed thoroughly before adding different concentrations of fumonisins standard (spiked). A 5 g of ground corn sample was used, mixed with 25 mL of 70% methanol and 30% water and then shaken vigorously for 3 minutes. The extract was filtered through a Whatman #1 filter paper to remove the solid material and the filtrate was then collected as the sample for analysis without further preparation. 

#### 3.5.2. Clean up using C-18 solid phase extraction (SPE)

For this procedure, samples extraction was conducted similar to the procedure described above. Sample filtrates were collected and cleaned using C-18 SPE (Waters, Milford, MA) following the procedure accompanying the SPE columns. The C-18 SPE column was first conditioned by sequentially passing 5 ml methanol and 5 mL water through the column. A 4 mL of sample filtrate was then passed through the column, followed by 6.0 mL deionised water. The fumonisins was then eluted from the SPE column by rinseing with 2.0 mL of methanol:water (70:30). The sample eluted was dried under nitrogen stream, re-dissolved in 0.01 M PBS pH 7.4 before 10 µL of samples used for the ELISA or immunosensor analysis.

## 4. Conclusions

An electrochemical immunosesnor based on gold working electrode using screen-printed sensor was developed for the detection and analysis of fumonisins in food samples such as corn. A monoclonal antibody against fumonisins was applied in this work in order to enable the analysis of fumonisins toxins (FB_1_ and FB_2_). A spectrophotometric immunoassay test was first developed in order to optimise the assay conditions and concentrations before moving the test on the surface of the electrochemical sensor. The final developed SPGE immunosensor exhibited a working ranges from 10–1000 µg·L^−1^ and the LOD was ~5 µg·L^−1^. This is well below the detection limit required for EU legislation of 2–4 mg·L^−1 ^of Fms (level required by official European Union). The sensor was also examined in extracted and cleaned up (using C-18 SPE columns) corn samples and showed very low matrix interference, high sensitivity and reproducibility. We have demonstrated that a competitive immunoassay for Fms, on an electrochemical screen-printed sensor device is capable of detecting fumonisins in corn samples and therefore has a great potential for on-site and rapid analysis. 
